# Preserved use of prior information under time constraints: an EEG study of action anticipation in expert athletes

**DOI:** 10.1186/s41235-025-00685-8

**Published:** 2025-10-26

**Authors:** Yingzhi Lu, Yujing Huang, Danlei Wang, Dongwei Li, Mengkai Luan

**Affiliations:** 1 School of Psychology, Shanghai University of Sport, 650 Qing Yuan Huan Road, Shanghai, 200438 China; 2 Key Laboratory of Sports Cognition Assessment and Regulation of the General Administration of Sport of China, Shanghai University of Sport, Shanghai, China; 3 Research Center for Exercise and Brain Science, Shanghai University of Sport, Shanghai, China; 4 Department of Psychology, Faculty of Arts and Sciences, Beijing Normal University, Zhuhai, China; 5 Beijing Key Laboratory of Applied Experimental Psychology, National Demonstration Center for Experimental Psychology Education, Faculty of Psychology, Beijing Normal University, Beijing, China

**Keywords:** Action anticipation, time constraints, EEG, Multivariate pattern classification, Expert athletes

## Abstract

Successful action anticipation in dynamic social environments, such as sports, requires the integration of prior expectations with observed kinematic cues. However, little is known about how temporal constraints modulate this integration process. In this EEG study, thirty-five expert basketball players completed a sport-specific prediction task in which both time constraints and prior cue availability were manipulated. Time constraints significantly impaired prediction accuracy, yet the behavioral benefit of congruent prior cues remained stable, indicating preserved strategic reliance on prior information. Multivariate pattern classification revealed that neural signals preceding action onset reliably distinguished trials with and without prior information in both time conditions, suggesting intact neural encoding of contextual priors regardless of temporal constraints. Time–frequency analyses further demonstrated increased parietal alpha synchronization during the preparatory phase and enhanced central mu suppression during kinematic processing under time constraints. These results indicate a shift toward more top-down, expectation-driven strategies when time is limited. Specifically, enhanced alpha activities may reflect compensatory top-down engagement to maintain predictive readiness, while mu suppression may support motor simulation when processing time is limited. Together, these findings underscore the brain’s flexibility in adapting anticipatory mechanisms to meet the demands of fast-paced and uncertain environments.

**Supplementary Information:**

The online version contains supplementary material available at 10.1186/s41235-025-00685-8.

## Introduction

The ability to anticipate others’ actions is fundamental to social interaction, enabling coordination, intention inference, and adaptive response in dynamic contexts. Effective anticipation integrates prior expectations and kinematic cues extracted from observed movements (Gredin et al., 2023; Kemmerer, 2021). This integrative process enhances interpersonal coordination and decision-making across domains, such as sports and communication. However, anticipatory processes in real-world contexts are seldom conducted under relaxed or unhurried temporal conditions. Instead, individuals often face substantial time constraints, requiring rapid prediction and decision-making based on incomplete or ambiguous information. It is therefore necessary to investigate how time constraints influence the integration of prior expectations and kinematic cues during action anticipation, and how this modulation is reflected in both behavioral performance and underlying neural processes.

Bayesian frameworks suggest that the brain combines prior expectations with sensory input to generate probabilistic predictions (Harris et al., 2023; Kilner et al., 2007; Knill & Pouget, 2004). In action anticipation, prior information—such as strategic tendencies—provides contextual cues, while kinematic features like posture and motion offer real-time evidence (El-Sourani et al., 2018; Koul et al., 2019). Optimal predictions require dynamic weighting of these sources based on their reliability. When sensory cues are ambiguous, prior expectations gain influence; when sensory evidence is clear, it dominates (Kelly & O’Connell, 2013).

Athletes are particularly adept at utilizing advance cues and maintaining performance under time-constrained conditions (Mann et al., 2007), suggesting that certain individuals may better preserve anticipatory function despite external temporal demands. However, Bayesian integration assumes adequate time and resources for processing, which may be compromised under constraints (Loffing & Cañal-Bruland, 2017). Limited time may hinder detailed kinematic analysis, shifting reliance toward heuristics or simplified strategies (Tenenbaum & Land, 2009). Such constraints might bias the integration process, leading to suboptimal weighting of prior and sensory cues and impairing the quality of anticipatory behavior. Building on this framework, we propose that time constraints modulate the integration process, increasing reliance on prior information while reducing sensitivity to kinematic detail. This modulation of information integration is expected to manifest in both behavioral performance and neural indices of anticipatory processing.

Although time constraints may disrupt optimal integration, cognitive systems adapt by reallocating resources and prioritizing low-cost information rather than discarding inputs altogether (Lavie, 2010; Shenhav et al., 2013). Under such constraints, individuals make strategic adjustments to maintain efficiency and predictive accuracy, often by shifting reliance or modifying processing timing. Time constraints likely simplify integration by favoring information that requires less cognitive effort. Prior expectations, operating through top-down mechanisms, offer an efficient route for guiding predictions by constraining the range of possible outcomes and reducing the burden on real-time sensory analysis (Friston, 2010; Summerfield & Egner, 2009). In contrast, processing dynamic kinematic cues demands more resource-intensive bottom-up mechanisms. Thus, under time constraints, individuals may accelerate the use of prior information to support anticipation, without entirely abandoning sensory evidence.

This strategic shift is expected to manifest neurally as enhanced preparatory activity following prior cue presentation. Two complementary neural signatures may index this anticipatory engagement. The contingent negative variation (CNV), a slow cortical potential emerging between a warning cue and an imperative stimulus, is associated with expectancy, attentional allocation, and motor preparation (Breska & Ivry, 2020; Hung et al., 2004; Wang et al., 2025). Increased CNV amplitude during the preparatory interval would indicate greater engagement with prior-based prediction under time constraints. Additionally, alpha oscillations may reflect internal belief updating and attentional control in the frequency domain. Recent findings suggest that higher alpha power encodes stronger Bayesian belief strength and more efficient allocation of attention (Haegens et al., 2011; Li et al., 2023). Thus, increased preparatory alpha activity may reflect a cognitive shift toward prior-guided anticipatory strategies when time constraints reduce the capacity for detailed sensory processing.

Beyond preparatory activity, time constraints may also influence the perceptual processing of kinematic cues. Some studies suggest that under compressed temporal conditions, individuals rely more on rapid perceptual sampling to maintain performance (Cisek & Kalaska, 2010; Heitz, 2014). Eye-tracking evidence further shows that experts display more efficient gaze patterns—shorter fixations and earlier target-focused saccades—when under time constraints, indicating adaptive reorganization of visual attention (Vickers, 2007). These findings raise the possibility that time constraints could enhance the extraction or weighting of kinematic information. However, neurophysiological studies suggest that sensorimotor simulation—often indexed by mu rhythm suppression—remains relatively stable and is shaped more by expertise than by short-term task demands (Del Percio et al., 2009; Denis et al., 2017; Fox et al., 2016). Experts consistently show stronger mu suppression when observing goal-directed actions, reflecting more robust internal action models, whereas novices show weaker or absent responses. Importantly, such mu modulation appears relatively insensitive to contextual variables like cue validity (Wang et al., 2025). Thus, whereas eye-tracking studies demonstrate how temporal constraints reorganize visual attention in the short term, mu suppression has often been considered a marker of expertise-related mechanisms of sensorimotor simulation, relatively less sensitive to immediate task manipulations. These findings suggest that time constraints shift anticipatory strategies toward greater reliance on top-down processing, with perceptual analysis of kinematic cues still playing a role.

Taken together, these considerations suggest that time constraints may influence action anticipation by modifying the efficiency and timing of information processing strategies, rather than fundamentally altering the perceptual extraction of kinematic cues. Specifically, temporal constraints may prompt earlier engagement with prior expectations and encourage a strategic simplification of integrative processes, while leaving core sensorimotor simulation mechanisms largely intact. Building on this framework, the present study examined how expert basketball players adapt their anticipatory processing under different temporal demands. Using a sport-specific prediction task, we combined behavioral and EEG measures to assess whether domain expertise supports flexible, context-sensitive anticipation. In doing so, we aim to clarify how the brain supports adaptive prediction in fast-paced, real-world environments, and extend existing literature by demonstrating that time constraints reshape the integration of prior information and kinematic cues in expert athletes, thereby advancing our understanding of the neural dynamics of anticipatory processing.

## Methods

### Participants

Thirty-eight expert basketball players (M_age_ = 20.38 years, SD = 1.33; 19 females), all members of the Chinese University Basketball Association (CUBA), were recruited for this EEG study. As CUBA is the premier national-level collegiate competition in China, all participants had extensive competitive experience in addition to their training background. All had trained ≥ 3 h/day, 5 days/week, for over 8 years, and held at least a second-grade National Player certification. None of the participants had a history of alcohol or drug dependence or any neurological or psychiatric disorders. Three participants were excluded due to excessive EEG artifacts, yielding a final sample of 35 right-handed players (Mage = 20.49, SD = 1.21; 17 females) with normal or corrected vision. All were naïve to the study purpose and provided written informed consent. The study was approved by the ethics committee of Shanghai University of Sport (Approval No. 102772022RT069).

### Stimuli

**Fig. 1:**
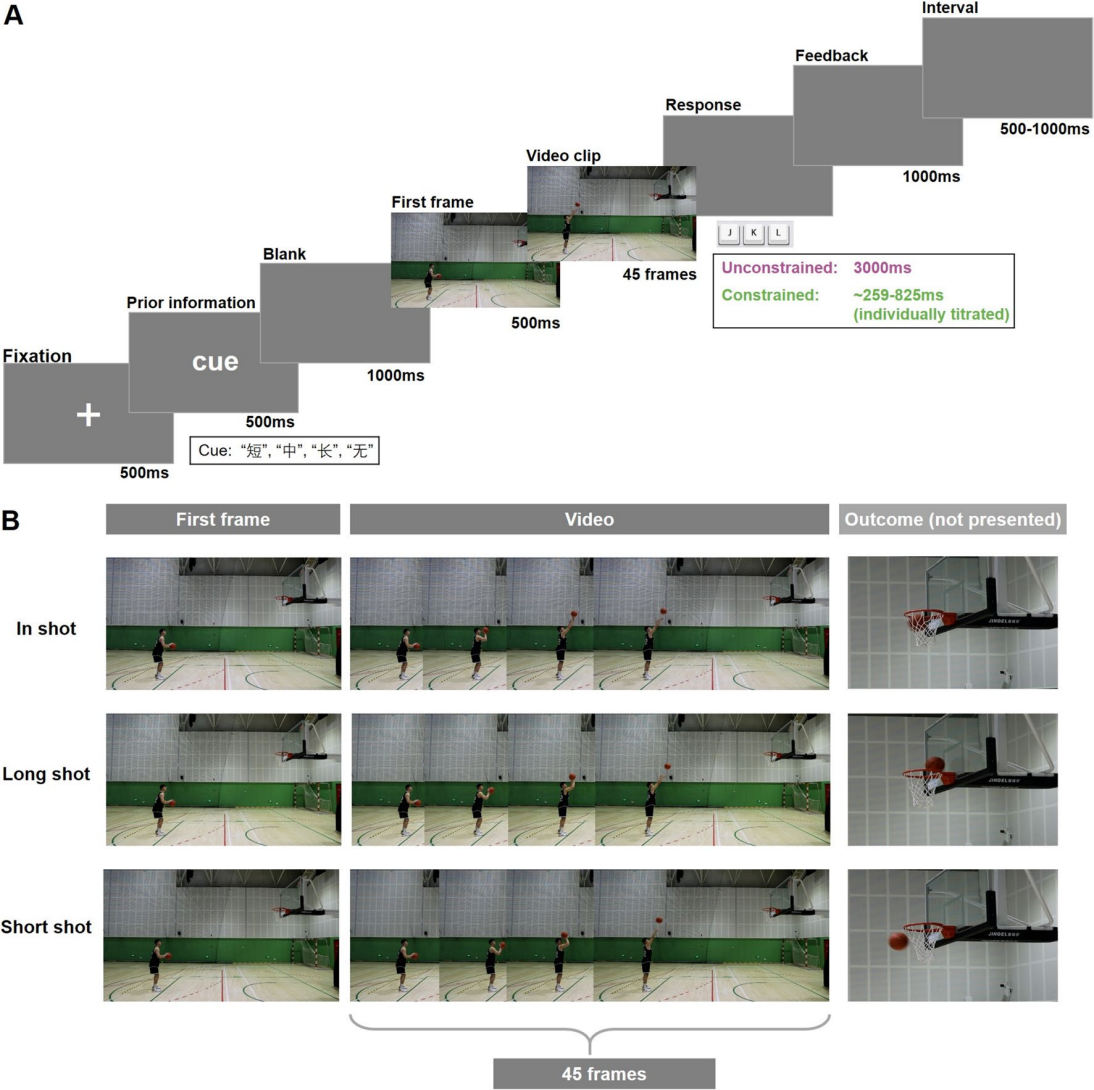
Experimental design and stimuli. **A** Trial sequences of the sport-specific action anticipation task. **B** Three types of free throws used as stimuli: in shot, long shot; and short shot (note: shot outcome was not presented in the stimuli)

The stimuli consisted of custom-recorded occlusion video clips filming two professional basketball players performing free throw actions (see Fig. 1B). Video footage was captured at 60 Hz using a Canon EOS R6 digital video camera positioned 6 m from a sagittal viewpoint. Two right-handed players, one male and one female, executed three distinct types of free throws: (1) in shots, where the ball entered the basket cleanly without touching the rim; (2) long shots, where the ball overshot the basket; and (3) short shots, where the ball fell short of reaching the basket. Each player completed 20 successful attempts for each shot type to ensure consistency. Video clips were edited using Adobe Premiere software to create occlusion sequences, spanning from 42 frames before ball release to 2 frames after release. Examples of the video stimuli are provided in the supplementary materials.

### Task and procedure

Participants were seated approximately 60 cm from a 17-inch monitor (1024 × 768 resolution, 60 Hz refresh rate) in a dimly lit, sound-attenuated room. They placed their right-hand fingers on the “J,” “K,” and “L” keys to indicate short, in, and long shots, respectively. The task included two within-subject factors: prior information (present vs. absent) and time constraints (present vs. absent). In the prior condition, a cue displayed one of three Chinese characters—“短” (short), “中” (in), or “长” (long)—indicating the likely outcome of the upcoming shot. In the no-prior condition, the cue displayed “无” (none). Participants were informed that the prior cues were generally valid, though the exact accuracy was not specified. To encourage reliance on both prior and kinematic information, cue-outcome congruency was set at 60%, well above the 33.3% chance level. Participants were instructed to predict the shot outcome based on both the prior cue (if available) and the observed kinematic information. This procedure was adapted from our previous work (Chen et al., 2024; Wang et al., 2025), which used a 53.3% congruency rate that influenced expert athletes’ responses but provided limited cue reliability. To improve cue effectiveness in the present study, the congruency rate was increased to 60%.

Each trial started with the presentation of a central fixation cross for 500 ms, followed by a 500 ms cue (either with or without prior information) and a 1000 ms blank screen. Then the first frame of the video clip appeared for 500 ms, followed by the full video clip. Participants were instructed to respond immediately after the video ended to minimize potential motor-related EEG artifacts. Feedback indicating “correct,” “incorrect,” or “too slow” was presented for 1000 ms. In the unconstrained condition, participants had up to 3000 ms to respond. In the constrained condition, a personalized response deadline was used, calculated as each participant’s median reaction time during the unconstrained trials (range: 259–825 ms, individually titrated). Late responses triggered “too slow” feedback, and the trial ended immediately. The inter-trial interval varied randomly between 500 and 1000 ms (see Fig. 1A).

The experiment consisted of two runs: one unconstrained run and one constrained run. Each run included four blocks (two prior, two no-prior) in an ABBA design. Each block contained 60 trials, with 1-min breaks between blocks. The unconstrained run was always conducted first to determine each participant’s baseline RT for deadline setting in the constrained run. A 5-min break separated the two runs to reduce fatigue. The same set of 60 video clips was randomly presented across blocks, and instructions were displayed at the start of each. After each run, participants completed the NASA Task Load Index (NASA-TLX; Hart & Staveland, 1988) to assess perceived cognitive workload. Before the main experiment, participants completed 12 practice trials (three per cue type) to familiarize themselves with the procedure.

### Behavioral data analysis

Trials with prior information were categorized into congruent (cue and outcome aligned) and incongruent (cue and outcome mismatched) conditions. Reaction time (RT) and accuracy were computed for each participant across three conditions: no-prior, congruent, and incongruent. Separate 2 (Time Constraints: unconstrained, constrained) × 3 (Congruency: no-prior, congruent, incongruent) repeated measures ANOVA was conducted for RT and accuracy. Notably, since participants were instructed to respond only after video offset, RTs may not reflect real-time anticipatory processing and were analyzed solely to validate the time constraint manipulation. Accuracy served as the primary indicator of anticipatory performance.

To assess how time constraints influenced information integration, we calculated the accuracy difference between congruent and incongruent prior information conditions under both time regimes. This cue-dependence index reflects reliance on prior information, with larger differences indicating stronger cue influence. To examine the consistency of integration strategies across conditions, Pearson correlation coefficients were computed to examine the relationship between cue dependence in the constrained and unconstrained conditions. Finally, paired-samples t-tests compared NASA-TLX scores across time conditions to determine whether perceived cognitive load differed between unconstrained and constrained conditions.

### EEG recording and preprocessing

EEG signals were recorded using Brain Vision Recorder (version 2.0; Brain Products GmbH, Germany) from 64 AgCl electrodes arranged according to the international 10–20 system, with a sampling rate of 1000 Hz. FCz served as the online reference and AFz as ground. Vertical and horizontal electrooculograms were recorded from electrodes placed below the left eye and at the outer canthi, respectively. Impedances were maintained below 5 kΩ throughout.

Offline preprocessing was conducted using EEGLAB (Delorme & Makeig, 2004) and custom MATLAB scripts. Data were re-referenced to the average of all electrodes and filtered with a finite impulse response bandpass filter (0.1–40 Hz). A notch filter (48–52 Hz) was applied to suppress 50 Hz line noise. Independent component analysis (ICA) was performed to identify and remove ocular artifacts, supplemented by manual inspection. Data were then downsampled to 500 Hz and segmented into 4.5 s epochs (− 2 to + 2.5 s relative to stimulus onset, defined as the onset of the first frame of the video stimulus). Baseline correction was applied using a 200 ms window before the cue (− 1.7 to − 1.5 s). Trials exceeding ± 100 μV were excluded, resulting in a 14.5% rejection rate. On average, 108.5 (prior) and 103.5 (no-prior) trials were retained under the unconstrained condition, and 101.2 (prior) and 96.8 (no-prior) under the constrained condition.

### ERP analysis

To assess cue-related preparatory neural activity prior to action observation, we analyzed the CNV. CNV amplitude was calculated as the mean voltage in the − 500 to 0 ms window relative to the onset of the first video frame, based on baseline-corrected data. This analysis focused on frontocentral electrodes (FC1, FCz, and FC2; see Fig. 3B), which are closely associated with expectancy-related processing (Kononowicz & van Rijn, 2014, Breska & Ivry, 2020). A 2 (Time Constraints: unconstrained, constrained) × 2 (Prior Information: prior, no-prior) repeated measures ANOVA was conducted on CNV amplitude. This analysis specifically targeted the influence of prior information on preparatory activity before the onset of kinematic cues. Although trials in the prior information condition included both congruent and incongruent outcomes, participants could not determine congruency at the time of measurement. Therefore, CNV amplitude was compared between prior and no-prior conditions irrespective of congruency.

### Multivariate pattern classification

Multivariate pattern classification was used to decode EEG signals associated with prior information under different Time Constraints conditions (Bae & Luck, 2018; Wang et al., 2025). Epochs extended from 200 ms before cue onset to 1500 ms after the first frame of the video clip. To improve decoding efficiency, EEG data were resampled to 50 Hz (i.e., one data point per 20 ms) and labeled by Prior Information condition (prior vs. no-prior). All electrodes served as features. A support vector machine classifier with error-correcting output codes (ECOC; Dietterich & Bakiri, 1994) was trained using MATLAB’s fitcecoc() and predict() functions. Fivefold cross-validation was repeated ten times with randomized splits. Classification accuracy was averaged across folds and iterations and smoothed with a five-point moving average. To assess whether decoding accuracy exceeded chance level (50%), a cluster-based permutation test with 10,000 iterations was conducted using one-sample t-tests across time points. Additionally, to compare decoding accuracy between constrained and unconstrained conditions, a second 10,000-iteration cluster-based permutation test was performed using paired-sample t-tests across time points. Multiple comparisons were controlled by identifying significant clusters across time points (for methodological details, see Bae & Luck, 2018).

Finally, to link decoding with behavior, mean decoding accuracy across above-chance time points was extracted for each participant in each Time Constraints condition. These values served as an individual-level indices of neural sensitivity to prior information. Pearson correlations were then computed between these values and behavioral cue-dependence index, analyzed separately for constrained and unconstrained conditions.

### Time–frequency analysis

EEG data were segmented from − 2000 to 2000 ms relative to the first video frame onset. Time–frequency decomposition was conducted using short-time Fourier transform (STFT) with Hanning tapers, estimating power from 2–30 Hz in 0.5 Hz steps. Event-related spectral perturbations (ERSPs) were calculated relative to a − 1700 to − 1500 ms baseline (preceding prior cue onset) and converted to decibels (dB) using 10 × log ratio transformation (Grandchamp & Delorme, 2011). Baseline correction was applied at the single-trial level.

We focused on the preparatory alpha activity (− 500 to 0 ms) and the post-stimulus mu activity (500 to 2000 ms). Alpha (8–13 Hz) power was analyzed at PCz and Pz, regions implicated in top-down attentional control and expectancy-related processing (Haegens et al., 2011, Li et al., 2023; see Fig. 4B). As with the CNV analysis, this window preceded kinematic cue onset; hence, alpha power was analyzed using a 2 (Time Constraints: unconstrained, constrained) × 2 (Prior Information: prior, no-prior) repeated measures ANOVA. Mu power (8–13 Hz) was analyzed at C3 and C4, electrodes over the sensorimotor cortex, reflecting motor simulation and action anticipation (Fox et al., 2016, Denis et al., 2017; see Fig. 5B). Mu power was analyzed using a 2 (Time Constraints: unconstrained, constrained) × 3 (Congruency: congruent, incongruent, no-prior) repeated measures ANOVA. In the time–frequency representations, decreases in power relative to baseline are shown in blue, and increases in red (see Figs. 4A, 5A).

To examine the relationship between neural oscillatory activity and action anticipation performance, correlation analyses were restricted to no-prior trials, as prior information did not yield significant main effects. This approach isolated the impact of time constraints without confounding effects of cue utilization. We calculated ΔERSP (constrained–unconstrained) for alpha and mu power and correlated these with corresponding ΔAccuracy values. Additionally, we tested whether ERSP values under each time condition predicted action anticipation accuracy, linking oscillatory dynamics to performance.

For all ANOVAs, effect sizes were reported as partial eta-squared $\left( {\eta_{p}^{2} } \right)$ values. When violations of sphericity were indicated by Mauchly’s test, Greenhouse–Geisser corrections were applied to adjust the degrees of freedom. Corrected values are reported throughout.

## Results

### Behavioral data

#### Subjective workload

**Fig. 2:**
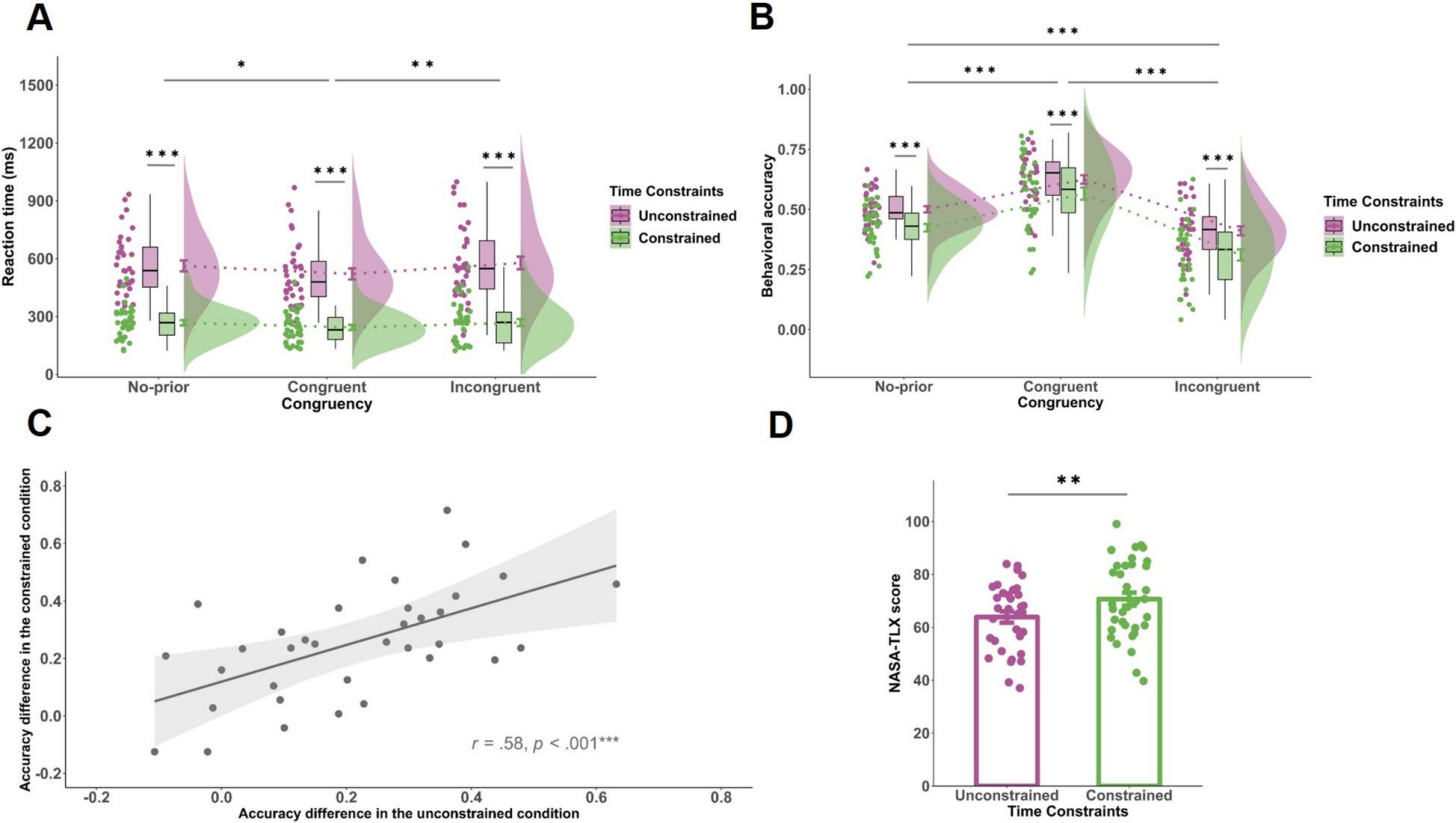
Behavioral results **A** Mean reaction time as a function of Time Constraints and Congruency. **B** Behavioral accuracy as a function of Time Constraints and Congruency. **C** Correlation of cue dependence (accuracy difference between congruent and incongruent trials) across Time Constraints. **D** NASA-TLX scores under unconstrained and constrained conditions. Significance levels are denoted as * *p* < .05, ** *p* < .01, *** *p* < .001

NASA-TLX scores were significantly higher in the constrained condition than in the unconstrained condition, *t*(34) = 3.75, *p* = .001, Cohen's *d* = 0.63, confirming that time constraints increased perceived task demands (Fig. 2D).

#### Reaction time

Participants responded significantly faster under constrained compared to the unconstrained condition, *F*(1, 34) = 256.30, *p* < .001, ${\eta }_{p}^{2}$ = .88. A significant main effect of Congruency was also observed, *F*(2, 68) = 7.30, *p* < .001, ${\eta }_{p}^{2}$ = .18. Bonferroni-corrected post hoc comparisons revealed that reaction times in the congruent condition were significantly faster than both the no-prior condition (*p* = .019) and the incongruent condition (*p* = .001). However, there was no significant difference between the no-prior and incongruent conditions (*p* = .98). The interaction between Time Constraint and Congruency was not significant, *F*(2, 68) = 1.11, *p* = .34 (Fig. 2A).

#### 3 Accuracy

A significant main effect of Time Constraint was observed, *F*(1, 34) = 29.49, *p* < .001, ${\eta }_{p}^{2}$ = .46, with overall accuracy being lower under constrained compared to the unconstrained condition. A significant main effect of Congruency was also found, *F*(1.52, 43.41) = 62.59, *p* < .001, ${\eta }_{p}^{2}$ = .65. Post hoc comparisons (Bonferroni-corrected) indicated that accuracy in the congruent condition was significantly higher than in both the no-prior and incongruent conditions (*p* < .001), and accuracy in the no-prior condition was also significantly higher than in the incongruent condition (*p* < .001). However, the interaction between Time Constraint and Congruency was not significant, *F*(2, 68) = 1.43, *p* = .25, suggesting that time constraint had a consistent effect across all congruency conditions (Fig. 2B).

Accuracy differences between congruent and incongruent conditions were positively correlated across constrained and unconstrained runs,* r* = .576, *p* < .001, indicating stable individual differences in cue reliance (Fig. 2C).

### ERP results

**Fig. 3:**
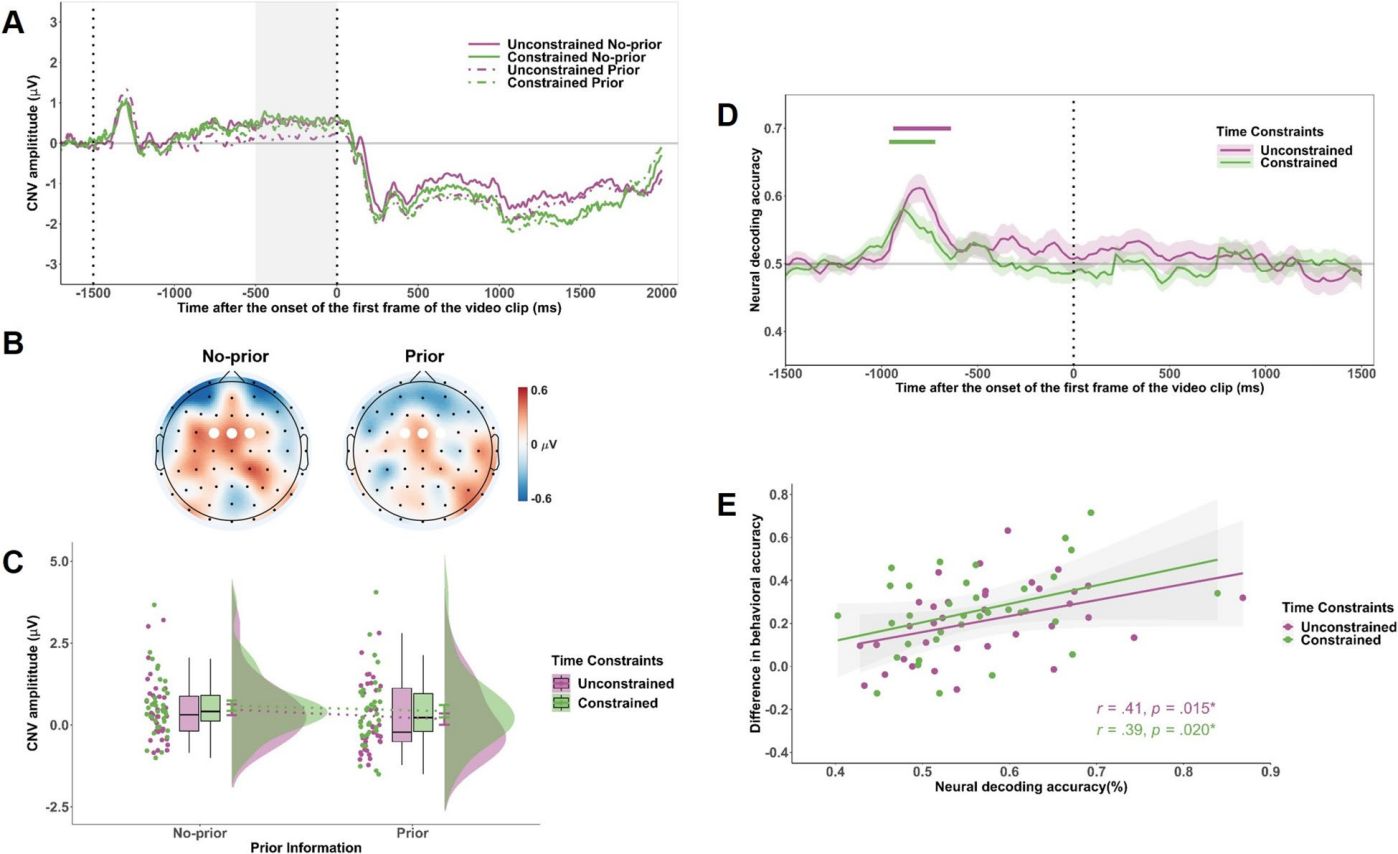
ERP and decoding results related to prior information processing. **A** Grand-average ERP waveforms at FC1, FCz, and FC2 for each combination of Time Constraints and Congruency conditions. The gray-shaded region indicates the CNV analysis window (− 500 to 0 ms relative to video onset). **B** Topographical maps showing CNV amplitude differences between constrained and unconstrained conditions (constrained minus unconstrained), separately for no-prior and prior information trials within the − 500 to 0 ms interval. White dots represent FC1, FCz, and FC2 electrodes. **C** Mean CNV amplitudes at FC1, FCz, and FC2 across Time Constraints and Cue Congruency conditions. **D** Temporal dynamics of decoding accuracy for classifying prior versus no-prior conditions under unconstrained and constrained conditions. Green and purple horizontal lines denote time windows where decoding accuracy significantly exceeded chance (50%) based on cluster-based permutation tests. **E** Correlation between individual mean decoding accuracy (in significant time windows) and behavioral cue dependence (accuracy difference between congruent and incongruent trials), analyzed separately for the unconstrained and constrained conditions. Significance levels are denoted as ** *p* < .05

Grand-average ERPs at FC1, FCz, and FC2 are shown in Fig. 3A. A repeated measures ANOVA revealed no significant main effect of Time Constraint, *F*(1, 34) = 1.73, *p* = .20, indicating that CNV amplitudes did not differ between the constrained and unconstrained conditions. The main effect of Prior Information was not significant, *F*(1, 34) = 2.56, *p* = .12, with comparable amplitudes between the prior information and no-prior conditions. No interaction was found, *F*(1, 34) = 0.35, *p* = .56 (Fig. 3C).

### Decoding results

Figure 3D presents the decoding accuracy for the constrained and unconstrained conditions. Multivariate pattern classification was applied to determine whether and when EEG activity could reliably distinguish trials with versus without prior information. The cluster-based permutation test based on one-sample *t*-tests revealed that in both the unconstrained and constrained conditions, decoding accuracy significantly exceeded chance level (50%) at specific time points, indicating that neural activity contained distinguishable patterns related to prior information processing. In the unconstrained condition, accuracy rose above chance at 560 ms after the prior cue onset and remained significant until 640 ms before the first video frame onset (*p*_*corrected*_ = .029). In the constrained condition, decoding accuracy was significantly above chance between − 960 ms and − 720 ms relative to the first video frame onset (*p*_*corrected*_ = .026). However, a cluster-based permutation test using paired-sample *t*-tests found no significant time windows where decoding accuracy differed between the constrained and unconstrained conditions. This suggests that although prior information was decodable under both conditions, time constraints did not modulate the temporal dynamics of prior information processing.

Pearson correlation revealed a significant positive correlation between decoding accuracy and the behavioral accuracy difference (congruent vs. incongruent trials) in both unconstrained (*r* = .41, *p* = .015) and constrained conditions (*r* = .39, *p* = .020), suggesting that stronger neural sensitivity to prior information predicted greater behavioral reliance on such cues (Fig. 3E).

### Time–Frequency representations

**Fig. 4:**
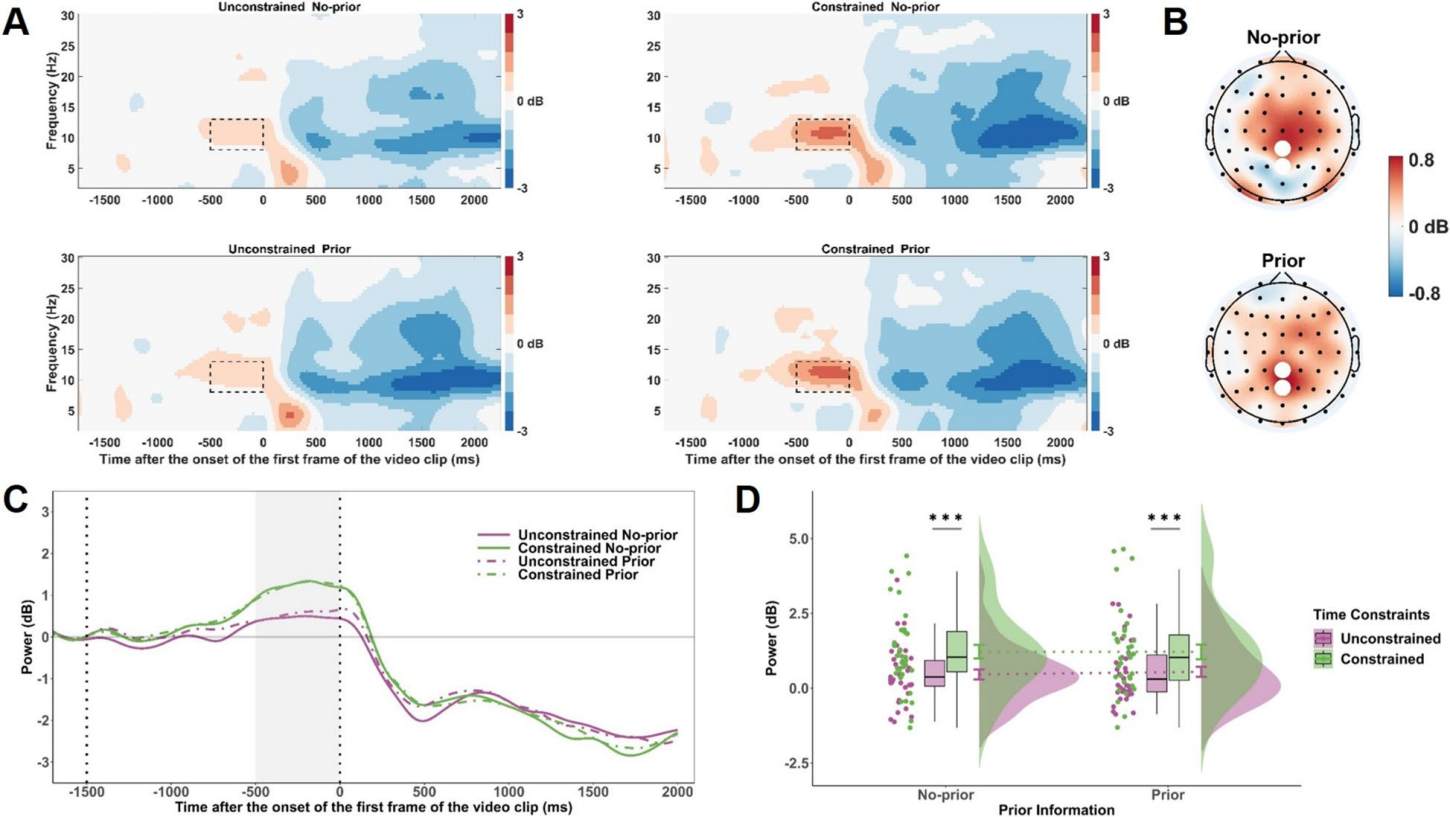
Time–frequency results for alpha-band activity during the preparatory period. **A** Time–frequency representations of alpha power (8–13 Hz) at PCz and Pz electrodes for each combination of Time Constraints and Congruency conditions. The black dashed boxes indicate the analysis window (-500 to 0 ms) relative to the onset of the video stimulus. **B** Topographical maps of the difference in alpha power between constrained and unconstrained conditions (constrained minus unconstrained), displayed separately for prior and no-prior conditions during the -500 to 0 ms time window. White dots represent PCz and Pz electrodes. **C** Grand average waveforms of alpha power over time at PCz and Pz electrodes, plotted separately for each combination of Time Constraints and Congruency conditions. The shaded gray region marks the analysis window (-500 to 0 ms). **D** Mean alpha power during the -500 to 0 ms window across Time Constraints and Congruency conditions. Significance levels are denoted as *** *p* < .001

**Fig. 5:**
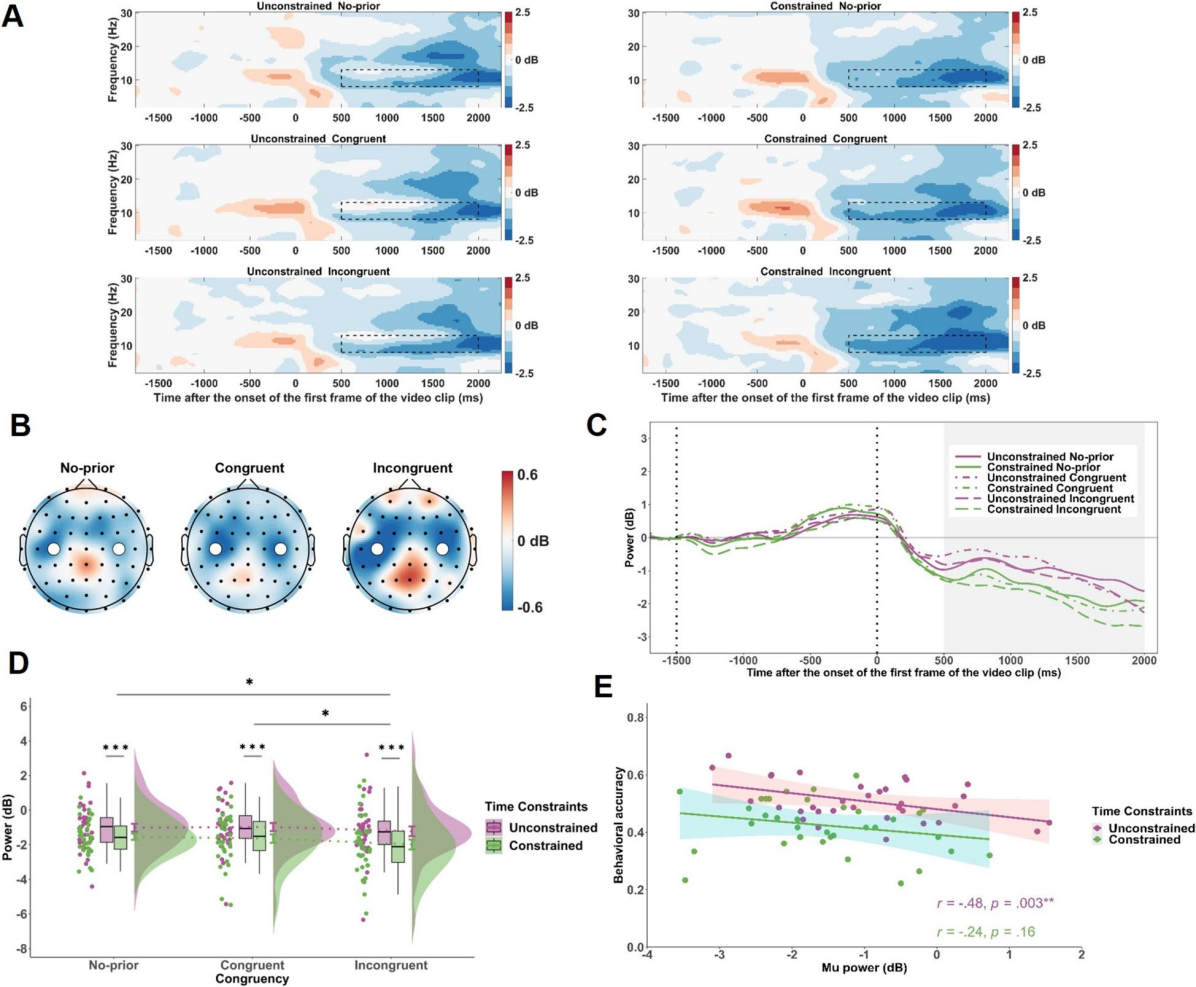
Time–frequency results for mu-band activity during the kinematic processing period. **A** Time–frequency representations of mu power (8–13 Hz) at C3 and C4 electrodes for each combination of Time Constraints and Congruency conditions. The black dashed boxes indicate the analysis window (500 to 2000 ms) relative to the onset of the video stimulus. **B** Topographical maps of the difference in mu power between constrained and unconstrained conditions (constrained minus unconstrained), displayed separately for congruent, incongruent, and no-prior conditions during the 500 to 2000 ms window. White dots represent C3 and C4 electrodes. **C** Grand average mu power waveforms at C3 and C4 across time, plotted for each combination of Time Constraints and Cue Congruency conditions. The shaded gray region indicates the analysis window (500 to 2000 ms). **D** Mean mu power during the 500 to 2000 ms window across Time Constraints and Congruency conditions. **E** Correlation between mu power and behavioral accuracy across participants, computed separately for the unconstrained and constrained conditions. Significance levels are denoted as * *p* < .05, ** *p* < .01, *** *p* < .001

Figures 4A and 5A display the time–frequency representations of alpha and mu power across prior information and time constraints conditions. The ANOVA revealed a significant main effect of Time Constraints on alpha power, *F*(1, 34) = 16.37, *p* < .001, ${\eta }_{p}^{2}$ = .33, with higher alpha power (greater synchronization) in the constrained versus unconstrained condition (Fig. 4C, D). No significant effects of Prior Information or interaction were found (*p*s > .11).

For mu power, there was a significant main effect of Time Constraints, *F*(1, 34) = 24.41, *p* < .001, ${\eta }_{p}^{2}$ = .42, with greater mu desynchronization (lower power) under time constraints (Fig. 5C, D). A main effect of Congruency also emerged, *F*(1.94, 65.98) = 5.06, *p* = .010, ${\eta }_{p}^{2}$ = .13. Post hoc tests showed stronger mu suppression for incongruent compared to congruent and no-prior trials (*p*s < .036), while the latter two did not differ (*p* = .98). No significant interaction was found, *F*(1.92, 65.21) = 0.47, *p* = .62, suggesting a stable congruency effect across time constraints.

Correlation analyses revealed that neither ΔMu power nor ΔAlpha power was significantly correlated with ΔAccuracy (*p*s > .43). However, a significant correlation was found between mu power and accuracy in the unconstrained condition (*r* = -.48, *p* = .003, see Fig. 5E), suggesting that stronger motor simulation supported better action anticipation when no time constraint was present. This relationship was absent in the constrained condition (*r* = -.24, *p* = .16), and alpha power showed no significant correlations with accuracy in either condition (*p*s > .71).

## Discussion

This study examined how time constraints influence the integration of prior expectations and kinematic information during action anticipation in expert athletes. While time constraints significantly impaired prediction accuracy, the behavioral benefit of congruent prior cues remained stable, suggesting preserved strategic reliance on prior information. Multivariate pattern classification revealed that neural signals preceding action onset reliably distinguished trials with and without prior information across both time constraint conditions. Time constraints enhanced preparatory alpha synchronization and increased mu suppression during kinematic processing; however, only mu suppression in the unconstrained condition was associated with better performance. In summary, the results demonstrate that time constraints alter the neural dynamics of anticipatory processing, even as behavioral reliance on prior cues was preserved.

Consistent with prior research on perceptual decision-making and motor performance (Kocher & Sutter, 2006; Vickers, 2007), our findings demonstrate that time constraints significantly impair action anticipation accuracy in expert athletes. While this decline is often attributed to the speed–accuracy trade-off (Bogacz et al., 2010; Heitz, 2014), anticipatory decisions in sports also rely on integrating prior expectations with unfolding kinematic cues (Abernethy et al., 2005; Cañal-Bruland & Mann, 2015). Thus, temporal constraints may disrupt performance not only by reducing response time, but also by interfering with perception, integration, or decision strategies. Three potential mechanisms may account for the observed performance decline: (1) impaired processing of kinematic information, (2) altered reliance on prior expectations, and (3) disrupted integration of prior and sensory information. In the sections that follow, we evaluate each possibility using convergent evidence from behavioral performance, subjective workload, and neural dynamics.

One potential explanation for the decline in anticipatory accuracy under time constraints is insufficient processing of dynamic kinematic information. Supporting this view, participants reported higher subjective workload on the NASA-TLX under time-constrained conditions, indicating that limited time not only reduced processing duration but also increased cognitive resource demands. Complementing this, stronger mu desynchronization under time constraints—typically reflecting greater motor simulation or mirror system engagement—suggests participants relied more on internal action representations to compensate for reduced bottom-up input (Denis et al., 2017; Fox et al., 2016; Hari et al., 1998; Muthukumaraswamy & Johnson, 2004; Pineda, 2005). However, this heightened sensorimotor activation did not improve performance. In fact, the typical association between mu suppression and better anticipation observed in the unconstrained condition disappeared under constraint. This dissociation suggests that although motor simulation mechanisms were more strongly engaged, the processing may have been shallow, fragmented, or poorly integrated. One possibility is that the activation of sensorimotor circuits reflects a default or compensatory response to high task demands, rather than an optimized information processing strategy. Alternatively, the increased reliance on motor simulation may have occurred at the expense of deeper perceptual analysis or more adaptive cue integration, leading to increased subjective load but reduced predictive utility. In either case, these findings highlight that under time constraints, greater neural engagement does not necessarily translate to better processing.

Another possible explanation is that time constraints may alter the decision strategy itself, specifically by reducing reliance on prior expectations. However, this interpretation is not supported by the current data. Behaviorally, participants consistently performed better on congruent than incongruent trials, regardless of timing condition, and no interaction was found between time constraints and congruency. This indicates that the influence of prior information on prediction accuracy remained stable. Electrophysiological results further reinforce this interpretation. Previous research has demonstrated that in sports contexts, effective directional cues can enhance CNV amplitudes, reflecting increased expectancy and motor preparation for the anticipated action (Hung et al., 2004; Wang et al., 2025). In this study, however, CNV amplitude did not differ across prior cue conditions or time constraints, indicating that the preparatory allocation of sensorimotor resources was not modulated by temporal pressure. Combined with the stable behavioral congruency effects, these findings suggest that athletes continued to rely on prior information to a similar extent and with similar preparatory engagement, regardless of the time available for response. Therefore, the decline in prediction accuracy under time constraints is unlikely to stem from reduced use of prior knowledge. Instead, the consistency of congruency effects and CNV amplitudes across conditions points toward a preserved anticipatory strategy, further narrowing the likely locus of impairment to later stages of the integration process.

Although MVPA revealed reliable decoding of prior versus no-prior trials under both time constraints, the differences in decoding onset were not statistically significant. Nevertheless, the consistent presence of decodable signals and numerical trends toward earlier engagement under constrained conditions suggest that athletes may initiate prior cue processing earlier when time is limited. This interpretation, though tentative, is consistent with the view that time constraints prompt anticipatory adjustments aimed at compensating for limited access to kinematic information.

Further support for anticipatory adjustments under time constraints comes from our findings on alpha-band activity. Specifically, we observed enhanced alpha synchronization in parietal regions during the preparatory phase under time-constrained condition. Alpha synchronization is commonly associated with top-down inhibitory control, reflecting the suppression of irrelevant inputs and facilitation of goal-relevant processing (Klimesch, 2012). In the context of action anticipation, heightened alpha activity may indicate a shift toward more internally guided prediction, allowing the brain to prepare for incoming kinematic cues based on prior expectations. This interpretation aligns with predictive coding frameworks, which propose that the brain continuously generates and updates internal models to reduce sensory uncertainty (Friston, 2005; Smith et al., 2021). Under time constraints, the reliance on such predictive models may become more pronounced, enabling faster, but less flexible anticipatory responses. This shift may also explain the observed increase in cognitive load and mu suppression, as cognitive resources are reallocated to reconcile prior expectations with constrained sensory input processing. Supporting this perspective, recent findings have shown that contextual priors can shape action understanding, even when kinematic information is degraded or ambiguous (Amoruso et al., 2019; Bianco et al., 2024; Magnaguagno et al., 2022). Our findings extend this understanding by demonstrating that temporal demands may enhance top-down modulation during anticipation, as reflected in increased alpha synchronization. In summary, although behavioral reliance on prior information remains stable, time constraints appear to alter the underlying neural strategy by promoting a more prediction-based mode of processing. This highlights the brain’s capacity to flexibly adapt its anticipatory mechanisms to meet the challenges of temporally constrained environments.

Several limitations of the present study warrant consideration. First, the time constraint manipulation targeted response execution time but did not limit stimulus presentation, leaving it unclear whether restricting sensory exposure would yield similar effects on anticipation. Second, prior cues were presented at a fixed validity level, preventing assessment of how time constraints influence the flexible weighting of prior information based on its reliability. Third, the study included only expert athletes without a novice comparison group, making it difficult to determine whether the observed anticipatory adjustments are specific to expertise or reflect broader cognitive adaptations. However, prior work from our group has shown that experts use prior information more stably and selectively than novices under perceptual conflict (Chen et al., 2024), suggesting that expertise may help preserve predictive strategies when cognitive resources are limited. Future research should compare athletes with varying experience levels to clarify how expertise shapes temporal flexibility in information integration. Moreover, incorporating complementary methodologies could provide converging evidence at multiple levels of analysis. For example, functional MRI could identify large-scale network mechanisms supporting anticipatory processing under time constraints, whereas non-invasive brain stimulation techniques such as TMS could establish causal contributions of motor and parietal regions to prior-based anticipation. Finally, the unconstrained condition was always conducted first to establish baseline response times. Although this approach enabled individualized calibration of time constraints, it also introduced the possibility of order effects, as participants may have gained more practice prior to the constrained condition. Nevertheless, the constrained condition consistently yielded declines in prediction accuracy and changes in neural dynamics, supporting the interpretation that these effects are attributable to temporal demands rather than practice alone.

## Conclusion

This study demonstrates that time constraints impair action anticipation in expert athletes, even though their behavioral use of prior information remains stable. Rather than disrupting predictive strategies, temporal demands appear to reshape the underlying neural processes, as indicated by increased preparatory alpha activity and stronger mu suppression during kinematic processing. These results suggest that athletes adapt by shifting toward more internally guided, expectation-based processing in preparation, and by engaging compensatory motor simulation during action observation. This neural adjustment reflects a flexible integration strategy under time constraints, offering insight into how the brain maintains predictive performance in fast-paced, uncertain environments.

## Supplementary Information

Additional file 1.

## Data Availability

The data and analysis code that support the findings of this study are available from the corresponding author upon reasonable request.

## Abbreviations

EEG: Electroencephalography
CNV: Contingent negative variation
RT: Reaction time
NASA-TLX: NASA task load index
CUBA: Chinese University Basketball Association
ICA: Independent component analysis
STFT: Short-time Fourier Transform
ERSP: Event-related Spectral Perturbation
dB: Decibel

## Declarations

### Open practices statement

The data and analysis code that support the findings of this study are available from the corresponding author upon reasonable request. The experiment was not preregistered.
